# O le Fau Gagana: A Samoan Mental Health Nurse in Aotearoa‐New Zealand

**DOI:** 10.1111/jpm.13104

**Published:** 2024-09-08

**Authors:** Taavale Ioana Mulipola

**Affiliations:** ^1^ Faculty of Health and Environmental Sciences Auckland University of Technology Auckland New Zealand

**Keywords:** mental health nursing, Pacific/Pasifika mental health, Samoan migrant woman, Samoan nurse

## Abstract

**Introduction:**

This article explores my journey as a Samoan woman who migrated as a young mother to Aotearoa‐New Zealand, completed nursing qualifications, later specialising in mental health nursing, and eventually completed doctoral studies. Since July 2023 I have been a Lecturer in the Department of Nursing, Faculty of Health and Environmental Studies, Auckland University of Technology.

**Methods:**

This article uses autoethnographic and narrative methods to collect data from my own life, to explore the experiences of Samoan people in the mental health system of Aotearoa‐New Zealand. Criteria for reporting qualitative research was used to prepare the paper.

**Findings:**

My story showcases the benefits of having both clinical and cultural understandings in the context of mental health care in Aotearoa‐New Zealand. The gap between the rhetoric and the reality of the ‘New Zealand dream’ for Samoan people mirrors the gap between policy and practice in relation to Pacific strategy plans for mental health care.

**Conclusions:**

By writing about my experiences, I aim to support better understanding of core concerns for Samoan people when they are engaging with mental healthcare services.

**Summary**

- Understanding cultural worldviews in westernised mental health care.

## 1 Introduction

This article is underpinned by the narrative of my own life, as one of the few first‐language Samoan speakers to achieve a Doctoral qualification and a career‐path academic job. This insider research adopts autoethnography (Bainbridge 2007; Ellis and Bochner 2000; Peterson 2015) as an approach for investigating Samoan mental health nursing (Foster, McAllister, and O'Brien 2006; Mila‐Schaaf and Hudson 2009), where my own life and experiences are used as sources of data, and where data collection and data analysis merge in ‘writing’ as a method (Richardson and St. Pierre 2018), including for Indigenous researchers (Stewart 2021). My story highlights the experience of being an indigenous migrant nurse working in a dominant westernised healthcare system.

Autoethnography is an emerging method in nursing research (Peterson 2015), especially in mental health nursing (Foster, McAllister, and O'Brien 2006). It is a method that applies data about self and contexts to acquire an understanding of the relationality between self and others in the same situation (Ngunjiri, Hernandez, and Chang 2010). In this case, I am exploring my dual positionality through both Samoan and mental health professional lenses. Autoethnography allows my voice to be heard, to bring new ideas and understandings to readers.

This narrative is told in the sections of my life journey from childhood in Samoa, to a career in mental health nursing, to becoming an academic at AUT. An account of my experience of fa'asamoa follows the first section about my life as a child in my ancestral village in Samoa. Together, these two sections provide a sense of a ‘Samoan worldview’ which is key to understanding the article. Also interspersed between the autobiographical sections are two vignettes or ‘short stories’ labelled Story 1 and Story 2. These stories are fictionalised but draw on my years of experience as a Samoan mental health nurse in Auckland, using a narrative method called ‘ethnographic fiction’ (Bruce 2014). Such narrative vignettes or stories aim for ‘verisimilitude’ meaning a true‐to‐life quality. Such ‘data‐stories’ aim to confront the reader with the reality and emotional impact of the research situation. Criteria for Reporting Qualitative research was used to prepare the paper (Tong, Sainsbury, and Craig 2007).

I compare myself to the fau tree. In Samoan tradition, the fau is an ordinary‐looking tree that was selected as the means by which to relay the mana (power, prestige) of the gods to our ancestors (Suaalii‐Sauni et al. 2018). This is why it is called the ‘fau gagana’ or ‘talking fau’. The wood of the fau is flexible and can be bent in a process called ‘so'ofau’ to provide the curves needed for building fale (Samoan houses). I am like the fau tree because I am an ordinary Samoan migrant woman, now living and working in Aotearoa‐New Zealand. My choices and resulting life experiences have equipped me with skills and knowledge to offer something that can benefit Samoan people in Aotearoa‐New Zealand. In this article, I will explore how I have held Western medical knowledge, clinical skills and nursing experience alongside my embodied understanding of Samoan cultural knowledge, values and practices.

## 2 Ole Amataga: The Journey Begins in Samoa

I was born and raised in the village of Vailu'utai in the rural south of Upolu Island in Samoa. I am the third eldest and the oldest female of 11 siblings. My father was a taro planter, and my mother was a homemaker. Our home was a simple, open Samoan fale (house), built by my father from wood and coconut leaves and surrounded by a small cocoa plantation.

Although the handmade wooden floor of my childhood home was rough and uneven, it was safe and comfortable. We ate largely from the sea and land, while our monetary income came primarily from my father's taro plantation, which funded our schooling. Each of my siblings had roles and responsibilities within our family, such as helping to weed the plantation, or harvesting cocoa after school and during school holidays. I still remember how proud I felt at about 5 years of age, when I first helped to collect water by carrying two medium pots from a nearby water well. I was born into a family with a simple way of life, based on coexisting harmoniously with nature, in which the values of close family relationships, connection and support were nurtured.

As the oldest girl in the family, I took on a lot of responsibilities from a young age. These included washing clothes, cleaning the house, cooking and looking after my younger siblings when my parents were not around. I also placed a lot of pressure on myself to do well at school, because I saw how hard my parents worked with their limited resources to pay for our education. The combination of my duties at home and my schoolwork was challenging at the time, but on reflecting now, I appreciate what I learned then about the value of hard work, compassion, determination, love and respect. While typical Samoan upbringings and childhoods have changed since that time, I have retained responsibility for my family's welfare, especially my mother. In Samoan culture, the female role is described by the phrase ‘o le pae ma le auli’ which means the woman is both the peacemaker (strong) and the counsellor (soft). This proverbial saying reflects a Samoan cultural expectation that females will play the role of negotiator, peacemaker and overall caretaker for the wellbeing of family members, as reflected in my own experiences. That way of life shaped who I am as a tama'ta'i Samoa (Samoan woman).

## 3 O le Fa'asamoa

In addition, my sense of self was shaped in my village. Within my village, there are a series of social systems in place. First, there is the fa'a Matai (the Matai system), which determines who holds the positions of village leadership and authority (Muaiava 2022). The matai or chiefs are responsible for leading, decision making and protecting the families making up the village. The matai is served and looked after by the members of the second social system of the village, the taule'ale'a (untitled men) (Suaalii‐Sauni et al. 2018). All young and old taule'ale'a are expected to be present when the matai council congregate for the fono a le nu'u (village meeting). During the fono (meeting) the taule'ale'a are not allowed into the house of matai; they sit at the back or outside of the fale fono (meeting house) and are responsible for preparing and serving food to the matai. The matai is either tulafale (orator) or ali'i (chief). The third social system is the faletua ma tausi, a group made up of the wives of the matai, in which the faletua are the wives of the ali'i, and the tausi are the wives of the tulafale (So'o 2006). The faletua ma tausi make up the women's council, which meets together monthly, or as needed. The women's council contributes to decision making and leads initiatives agreed upon by the village fono. Members of this group includes all women who are married to matai holders, both those of the village and those from other villages.

The final social system of the village is the aualuma (women's guild). All women of the village, both young and old, belong to this group. Leaving school is the criterion to determine when a girl becomes a woman, whereupon she joins this group. The core function of the aualuma is to look after the health and well‐being of the village. Together with the faletua ma tausi, this group assists the national health services with initiatives such as immunisation, dispensing special medication, conducting well‐child and community health checks and monitoring the houses of the village for hygiene and sanitation. The members of this group are only those women who belong to the village by ancestry.

Growing up in my village with these different social systems operating has shaped my worldview in terms of my deep level of understanding and acceptance of how people behave towards each other, and where I fit within Samoan society. This worldview has engrained within me the essence of the relational way of village life in which each person and environment are equal and complementary to each other. The term va fealoaloa'i refers to the cultural protocols and ethics that protect the sacred boundaries between each interpersonal relationship and between these different social systems in the village. It is a way of life that celebrates human relationships, communication, caring, compassion and respect and is underpinned by the values of alofa (love) and fa'aaloalo (respect) (Va‘ai 2014). Indeed, this worldview is the core foundation of Samoan collectivism and holistic concepts of health and well‐being.

## 4 The New Zealand Dreams

Like most people in Samoa, I heard people talking about New Zealand all my life. It was always described as paradise on earth and has been a dream destination for many Samoan families. It was described as a beautiful place where everyone speaks English, the lifestyle was easy, and there was more money than we have at home. I often heard these comments from family and village members when they returned home from visiting New Zealand. I remember my parents, like most others, seeking opportunities from family or friends who were already settled in New Zealand to sponsor us. Samoan people who came back after living there for some time appeared and acted differently; their skin was fair, and they seemed to have a lot of money. Their children were speaking English and wearing nice clothes. When I was young, I envisioned this faraway place as magical and fun.

As I grew older, I kept working hard at school and was ultimately rewarded with academic success by achieving School Certificate (the New Zealand qualification) in English, Biology, Geography and awarded Dux at the end of Year 11, which was as high as my school went. My achievements allowed me to continue for one more year in the Catholic Senior School in Apia, completing Sixth Form Certificate. When I graduated, I began working as a bank teller at the Bank of Western Samoan now known as the Auckland and New Zealand Bank (ANZ) in Apia, where I met my husband. We were married and had four daughters born between 1992 and 1998. The youngest was 1 month old when I resigned from ANZ and we boarded a plane to come to Aukilani (Auckland).

When my family and I arrived in Aotearoa‐New Zealand it was a culture shock. We were confronted with ways of life that were the opposite of what was familiar from home. Far from the village in which I grew up, I was now living in a suburb with people from many different backgrounds and cultures. Families were separated into nuclear families, which was hard to accept. There were many moments when I had to remind myself why we left Samoa—to seek better opportunities for our children—and therefore why we needed to make it work. Once all the children were at school, I enrolled in a Bachelor of Nursing at Manukau Institute of Technology in Otara, South Auckland.

## 5 Becoming a Nurse in Aotearoa‐New Zealand

I decided to study nursing to get money for my family. After completing one semester of foundation studies, I was accepted into the nursing degree programme. Studying nursing was an opportunity to discover personal abilities I had never used. I discovered my interest in public speaking, leadership, team work and ongoing learning. Nursing exposed me to different areas of health care, and I became interested in mental health. Nursing also made me aware of the challenges Samoan and Pacific people face in health care (Kapeli, Manuela, and Sibley 2020), and eventually it became more than simply a way to earn money. Nursing developed into a passion to serve, and advocate for, Samoan healthcare consumers.

## 6 Story 1: ‘You Should Be grateful’

At Age 19, Sione is a strong and handsome young man, but has been causing all sorts of problems for himself and his family because of his angry outbursts. These started happening when he was about 12, soon after he came to live in Auckland with his family. Lately, his outbursts have been becoming increasingly violent and scary, interspersed with bouts of apathy, when he refuses to leave his room for days on end.

Mental health nurse Sina is meeting with the psychiatrist, Dr Whyte, to discuss Sione's care.

‘Sione agrees to remain under the Mental Health Act—he signed the form, and he has a copy’ says Dr Whyte, with an air of satisfaction, knowing that he has completed the required paperwork process.

Sina asks, ‘Did Sione understand what he signed?’

‘Oh, he can speak English, so he should be okay’ replies Dr Whyte.

Sina keeps going. ‘Did you involve his mother, because he lives with her?’

Dr Whyte responds ‘He's been with the service for some years and his mother is familiar with the process. Anyway, the family are very hard to engage with’.

Sina wonders about the family's reasons for not engaging—is it due to a language barrier, or does it have more to do with Dr Whyte's approach? Does he know about the Pacific health models that student nurses learn about in their training? Of course not, how would he, since they are not visible in practice.

‘What other means did you use to engage with them? Did you involve the Pacific service?’ Sina is frustrated. Dr Whyte is annoyed.

‘Placing Sione under the Act would ensure his compliance with treatment and avoid the need to involve the police if he gets unwell’ he points out.

‘Well, I can understand your point, but you should at least inform his mother’. Sina is thinking about the numerous conversations she has had with health professionals about this. She thinks to herself, ‘Why do I have to keep having this conversation about cultural practices for Pacific consumers? Isn't that our model of care? Why do I have to keep reminding people about this? I should be compensated’.

Sina speaks with Sione in the Samoan language about the form. ‘Oh, they gave it to me to sign’ he tells her.

‘Did you understand it?’ ‘No, they said just sign’. Probing a bit further, Sina finds out that Sione does not know how to read written English. He can speak basic English but has learning disabilities and has never been successful in learning to read. Sina becomes concerned, wondering how they expect him to understand his diagnosis, the medications he is taking and side effects.

She meets with Dr Whyte again. ‘I need to advise you that Sione does not know how to read and understand the English language’.

Dr Whyte responds, ‘Thank you for letting us know, that is awesome, after all we are providing holistic quality care—that's our approach. Besides, we are short staffed and there are not enough Pacific professionals working here. We are trying our best to provide quality care—you people should be grateful to get these services for free’.

Despite his frequent references to ‘care’ it is apparent to Sina that Dr Whyte does. not. care.

### 6.1 The Journey Continues

Following my new‐found passion for nursing as service to my people, I aimed to build up my nursing career by enrolling in part‐time postgraduate studies, one paper per semester, slowly but surely completing first a postgraduate certificate, then a diploma, and finally a Master of Nursing (MN) degree. Postgraduate study required all my energy and courage, as had all of my studies since embarking on the nursing pathway. I had to work twice as hard to read and write in English, my second language, especially in the academic genres of advanced study.

During my career as a mental health nurse, I received positive feedback from my managers on many occasions. ‘You are a great nurse’ said my manager after one team discussion. I thought she must have liked my contribution at various staff meetings, or maybe she was aware of my successful consumer outcomes? For example, I was able to persuade Pacific clients to accept their medications more successfully than my Palagi (White/Pākehā) colleagues. ‘We're lucky to have you’ said my nurse colleague, after one such outcome.

## 7 Story 2: The Work of the Pacific Mental Health Service

Fale is 35 and has recently migrated from Samoa to live in Auckland with her husband and two children. She was pregnant when she arrived in New Zealand and delivered her baby at Middlemore Hospital. But she has not been sleeping well and keeps hearing the voice of her grandfather, reminding her about her siblings and people back home. She is finding it hard to form an attachment to her new baby, which the nurses in the maternity ward notice, and refer her to the maternal mental health team. Fale is given a diagnosis of post‐partum psychosis, for which she is prescribed antidepressant medication, sleeping tablets and therapy sessions with the psychologist. But there is a strong stigma associated with mental illness in the Samoan community, and Fale is reluctant to take her pills. Also, Fale's husband is finding it hard to accept that she is unwell, since she was fine when their two older children were born back in Samoa. But there she had the support of her mum, sisters and the rest of the extended family, who all lived close by, and gave her plenty of help with her babies.

Because she is a second‐language speaker of English, Fale is referred to the Pacific mental health service, who provide her with education in her own language about her illness and help her understand her treatment and contributing lifestyle factors to support her mental health and well‐being. The service also provides support for her husband, to help him understand and support Fale and what she is going through. The service also involves the local Samoan community support worker, who can help Fale and her husband to access financial and social support with housing, and to help the family bring Fale's mum over to New Zealand for a visit. Her sister, who is already living in Auckland, is also included in the care plan, and over time becomes more involved in Fale's life and family.

After 3 months, Fale is slowly recovering, and becoming able to make her own choices and care for her own well‐being, as well as for her husband and children. She has developed stronger connections within her local community and is more aware of where to go for help and to access services when she needs them. Through this experience, her whole family has become more aware of mental health issues and services available.

### 7.1 Living the Dream

Three years after completing my MN, I went back to study again, this time undertaking doctoral study. My thesis research investigated the experiences of Samoan consumers in the mental healthcare system. My perseverance paid off when I eventually passed my doctoral oral examination with no corrections required to my thesis (Mulipola 2023; Mulipola, Holroyd, and Vaka 2023). My doctoral qualification was a source of pride, not only for myself personally but also for my whole family and community.

My achievement was celebrated in true Samoan style. More than 100 of my extended family and friends came to the Fale o Samoa in Mangere, including my siblings who travelled from Samoa and Australia for the occasion. I looked around and saw happiness, pride and love on the faces of the people who were there for me. I heard family members saying, ‘Malo le fa'aea aiga’ (Good on you for elevating the status of our family) and ‘Ua tā fia Falealili fua’ (I want to be like Falealili—a famous village in Upolu). My sister told me ‘Thank you for your achievement, I am somebody now, because of you’ which was humbling for me to hear. I had taken advantage of the significant opportunities offered in this country to grow and prosper, but faced many barriers and challenges along the way, which I had overcome with perseverance, hard work and determination. It was overwhelming to realise how much my achievement meant to others. It seemed like I had achieved my version of the ‘New Zealand dream’ not only for myself and my family but also for the whole New Zealand Samoan community.

The health teams I worked in were proud of their strategic plans for increasing Pacific leadership. Yet repeatedly, over the years, my applications for leadership roles were declined. I was told they were looking for more seniority, more experience, more specialist medical qualifications. In one case, I was asked to induct the medically qualified person who was displacing me in my scoping role. There always seemed to be subtle negative forces pushing against me, and I wondered to what extent it was because I am Samoan. I was the odd one out, the only one from my peer group to have come this far, yet it was as if I would never be good enough for those who hold power in the health system. I thought about how many Pacific and migrant nurses must be discouraged by these subtle forces from trying to advance their careers.

At the same time, despite my personal migration success story, the reality of life for immigrant Samoans in Aotearoa‐New Zealand is nothing like the New Zealand dream I grew up with, which pushes so many Samoans to migrate. Immigrant Samoans in New Zealand have life‐long obligations to send money to support family back home, which can place them under significant financial pressure. This is one reason why Samoan parents might encourage their children to leave school and work to help bring in money, rather than supporting them to carry on with further education. Younger generations of Samoans are growing up in Aotearoa‐New Zealand not understanding or speaking Gagana Samoa. For various reasons, many immigrant Samoans lose touch with their fa'asamoa.

Most Samoan stories about life in New Zealand revolve around material possessions and monetary success; the ability to buy new houses and cars, to upgrade the family's living standards compared to others in the village. But the New Zealand dream has a dark side, relating to the loss of familiar people, language and the social support of the old ways in the traditional villages. For many Pacific people, factors associated with migrating to New Zealand result in psychological distress and ill health. The dream of migration from our island homes to our adopted country Aotearoa‐New Zealand is for our family and children to prosper and be healthy, not to continue to suffer the worst health outcomes in the country, including psychological distress. My experiences show that although family wealth is important, Samoans must remain aware of the psychological damage that migration can cause to us as Samoan adults and to our children. The detrimental consequences for individual and collective well‐being outweigh the possible material gains on which we tend to focus.

As a result of negative Pacific health statistics, every hospital and health provider in the country has policies about offering appropriate levels of service to Pacific people. The two stories included above can be seen as a story of failure and a story of success for Samoan mental health care. Topics like culturally sustaining practices and Pacific health are standard in every tertiary course for nursing and allied health professions. People like me are employed to teach these topics—roles like mine exist because my Palagi (white) colleagues cannot teach these topics. Yet clearly, I cannot teach nursing students to ‘be’ Samoan, nor overcome the collision and contradiction between cultural and medical care in the health system of Aotearoa‐New Zealand. How will sharing my culture with non‐Samoan nursing students force them to examine their own subconscious assumptions, or does this approach simply leave those assumptions unexamined and allow the status quo to continue?

The reality of life for Samoans in New Zealand contributes to their negative health statistics; the response of the health system is to devise policies and plans for increasing the number of Pacific people at all levels of the health workforce (Kapeli, Manuela, and Sibley 2020). Yet my own experience and those of others like me is that we are not good enough for leadership roles. We are needed but must remain in our place.

## 8 Conclusion

Like the fau tree, my life has been ordinary, but built on strong foundations. My strength comes from my fluency in the Samoan language and my innate understanding of the values of alofa and fa'aaloalo. I know who I am, and where I come from. This cultural foundation has given me tenacity and flexibility like the fau, so that I can bend and withstand the challenges of migration and higher education, seeking after medical knowledge and qualifications. It enables me to develop a critical stance from which to investigate and improve the provision of mental health services for Samoan consumers.

In terms of the ‘New Zealand dream’ held by many Samoans, there is a gap between the utopian rhetoric and the harsh reality of life as a Samoan immigrant in Aotearoa‐New Zealand, most residing in large urban centres. Life is grinding, not beautiful; beset by problems that make life back home seem like an idyllic memory. This gap between the dream and the reality of life in New Zealand is analogous to the gap in relation to the place of Pacific cultures in health provision and leadership. This research has given an insider Samoan account of the complex relationships, paradoxes and contradictions that lead to, and maintain, these gaps.

## Conflicts of Interest

The author declares no conflicts of interest.

## Data Availability

The data that support the findings of this study are available from the corresponding author upon reasonable request.
